# Mutual Recognition: Empathy as the Foundation of Community in Dementia

**DOI:** 10.1093/geroni/igab046.1535

**Published:** 2021-12-17

**Authors:** Peter Costello

**Affiliations:** Providence College, Providence, Rhode Island, United States

## Abstract

This paper explores the challenges of developing a healthy, genuine community as some of its members experience cognitive decline or dementia. I draw upon philosophical discussions on community (Stein, 2000) and Husserlian empathy (1931;1939) to identify these challenges. First, community is organic; it relies on the differentiated roles of individual members to remain healthy. The ability to recognize the contribution of each member is essential for its health. Second, dyadic relationships may similarly be healthy or waning depending on the presence or absence of mutual empathy. Empathy is embodied. Persons living with dementia (PLWD) need to experience being recognized as persons, in person, in order for dyadic relationships and communities to thrive. As such, some communities may become unhealthy in the absence of mutual recognition. In these instances, careful interventions, e.g., through shared experiences and embedded memories, may be required to promote the well-being of the community and its members.

